# Assessing the Impact of Nigerian Government Responses to Armed Banditry in Northwest Nigeria, 2017–2025

**DOI:** 10.12688/openresafrica.16703.2

**Published:** 2026-10-05

**Authors:** Aitse John Zinna, Dimas Garba

**Affiliations:** 1 Department of Political Science and International Relations, Nile University of Nigeria, Abuja, Federal Capital Territory, Nigeria

**Keywords:** armed banditry; state fragility; security sector reform; human security; Responsibility to Protect; government effectiveness; community trust

## Abstract

**Background:**

Armed banditry in Northwest Nigeria has evolved from rudimentary cattle rustling into a coordinated security crisis claiming thousands of lives and displacing over 1.3 million persons between 2017 and 2025 according to various reports.

**Objective:**

This study assesses the impact of Nigerian government responses to banditry during this period, examining whether interventions measurably reduced violence, strengthened state authority, restored human security, and addressed structural drivers of conflict.

**Methods:**

A mixed-methods design combined quantitative analysis of survey data from 400 respondents across Zamfara, Katsina, and Kaduna states, using descriptive statistics, one-sample t-tests, one-way Analysis of Variance (ANOVA), chi-square analysis, and multiple linear regression, with qualitative thematic analysis of thirty-eight key informant interviews and cross-state comparative analysis of secondary security data (2017–2025).

**Results:**

Quantitative findings confirm that armed banditry materially undermined national security across all measured dimensions (composite mean = 4.31, p < 0.001). Government responses were acknowledged as active (mean = 3.52, 61.3% agreement) but perceived as failing to address root causes (mean = 2.01, 76.1% disagreement) and as ineffective in protecting lives and property (composite mean = 2.61, p < 0.001). Multiple linear regression identified community trust (β = 0.247), community consultation (β = 0.194), rebuilding programmes (β = 0.187), and inter-governmental collaboration (β = 0.165) as the strongest predictors of perceived security effectiveness (R ^2^ = 0.895).

**Conclusion:**

Government responses have been predominantly reactive and militarized, achieving temporary tactical gains without sustainable civilian protection or structural transformation. Effective counter-banditry governance requires a strategic shift toward people-centered, integrated, and community-anchored approaches.

## 1. Introduction

Between 2017 and 2025, armed banditry transformed from a security concern in the fringes into one of the most lethal non-ideological internal conflict confronting the Nigerian state. Across the Northwest geopolitical zone, comprising Zamfara, Katsina, Kaduna, Sokoto, Kebbi, Jigawa and Kano states, criminal networks that originally operated as loosely organised cattle-rustling gangs evolved into structured armed entities conducting mass abductions, large-scale village raids, attacks on security formations, and systematic extortion of rural economies. The human cost has been severe: independent assessments indicate that banditry accounted for approximately 13,485 deaths between 2010 and May 2023 ( Sadiq & Ojewale, 2023), with several others being displaced. Between 2024 and 2025, an estimated 4,700 people have fallen victims to attacks across 70 local government areas ( Tijjani, 2025). An estimated that ₦2.2 trillion was paid as ransom nationally between 2023 and 2024, with the Northwest accounting for 54.5% of ransom payments and 63.5% of kidnapping incidents ( National Bureau of Statistics, 2024, as reported in TheCable, 2024).

The Nigerian government both at federal and state levels have responded with a range of kinetic and non-kinetic measures. At the federal level, this encompassed major military operations including Operations Sharan Daji, Hadarin Daji, and Whirl Punch, the deployment of air assets, and the formal designation of bandit groups as terrorist organisations in November 2021 ( Federal Government Gazette, 2021). At the state level, responses included amnesty programmes, peace dialogues, vigilante formalisation, and community policing initiatives, among others. Yet despite these efforts, the crisis has proven stubbornly resilient. Bandit networks have fragmented into smaller, mobile cells, adapted to evade military operations, crossed porous borders into Niger Republic ( Garba, 2021; Okoli & Abubakar, 2021) and particularly from 2024 onwards they resumed mass abductions of schoolchildren in Kebbi and Niger states ( UNICEF, 2025).

This persistence raises fundamental questions about the effectiveness (effectiveness defined in Section 3.2), coherence, and sustainability of Nigeria’s counter-banditry governance. Existing scholarship has explored the causes, typology, and dynamics of banditry in considerable depth ( Garba, 2021; Okoli & Abubakar, 2021; Sarkin Gulbi et al., 2024; Akinyetun, 2022; Abdullahi, 2022). Less attention has been devoted to rigorously assessing the measurable impact of government responses, whether interventions reduced attack frequency and severity, strengthened state authority, restored socio-economic stability, and addressed the structural drivers of conflict. This study fills that gap.

**Figure 1.**
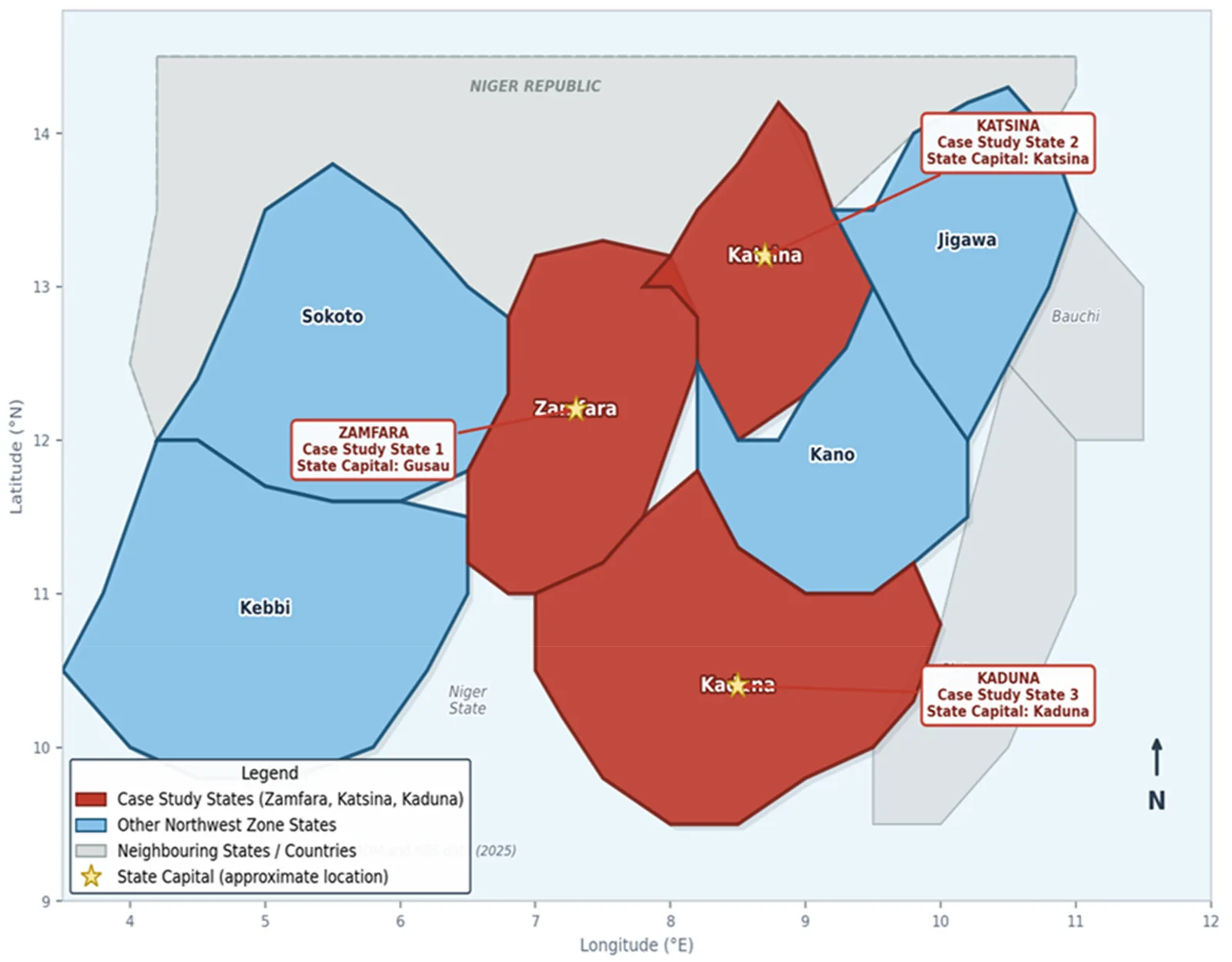
Map of Northwest Nigeria Showing Case Study States. *Source*: Author’s compilation based on GADM administrative boundary data and NBS geospatial records (2025).

The research poses four core questions: What major policies and strategies did government deploy to combat banditry in the Northwest between 2017 and 2025? To what extent did these responses reduce violence, improve security, and restore socio-economic stability? How did affected communities and stakeholders perceive and experience government interventions? And what lessons can be drawn to inform more sustainable and effective counter-banditry approaches? The study draws on State Fragility Theory ( Rotberg, 2004), the Human Security and Security Sector Reform Paradigm ( UNDP, 1994; OECD, 2007), and the Responsibility to Protect (R2P) doctrine ( United Nations General Assembly, 2005, paras 138–139) to structure both its analytical framework and its evaluation criteria.

## 2. Literature review

### 2.1 The evolution and nature of banditry in Northwest Nigeria

Armed banditry in Northwest Nigeria defies simple categorisation. It combines elements of organised crime, quasi-insurgency, and socio-political rebellion, shaped by decades of governance neglect, resource competition, and the progressive erosion of traditional conflict-management institutions and weak state presence in peripheral areas. Historically, armed banditry initially manifested as cattle rustling and localized violence. Over time, however, it transformed into organized armed criminality due to the proliferation of small arms, commercialization of violence and state neglect ( Okoli & Okpaleke, 2014). This phenomenon underwent structural transformation from approximately 2011 onwards, when criminal networks began establishing territorial control over ungoverned forest spaces, acquiring military-grade weapons, and systematising ransom-based economies ( Garba, 2021; Amadi & Onu, 2026). By 2017, the crisis had achieved the scale and sophistication that characterises asymmetric armed conflict: coordinated multi-front raids, hierarchical command structures, cross-border arms supply chains, and parallel taxation of rural communities ( Okoli & Abubakar, 2021).

The following structural drivers have been consistently identified in the literature; Persistent poverty and youth unemployment, with the Northwest recording poverty rates exceeding 70% and youth unemployment surpassing 45% between 2017 and 2024 as stated by the National Security Adviser – Nuhu Ribadu in 2024 ( Maruf, 2024), this has created conditions of material desperation that criminal networks exploit. Another Driver is the environmental degradation and climate-induced displacement have intensified farmer-herder competition over shrinking arable land, and the proliferation of small arms through transnational trafficking networks linked to Libya, the Sahel, and Niger and Chad has dramatically lowered the cost of organised violence: over 70 per cent of seized weapons in the Northwest originate from illegal transnational routes ( Amadi & Onu, 2026; Bassey et al., 2025).

### 2.2 Evaluations of government response

Existing studies assessing government response have arrived at broadly similar conclusions. Aina et al. (2023) evaluated Nigeria’s military “shock and awe” strategy and found that while deployments temporarily degrade bandit mobility, fragmentation, terrain familiarity, and criminal adaptation blunt deterrence effects without integrated governance measures, Fallata et al. (2024) found government actions to be reactive and fragmented, with limited community engagement and weak rural policing undermining intelligence capacity. Abdullahi (2022) argued that coercive approaches without parallel socio-political remedies exacerbate grievances and fuel displacement, advocating for people-centred security combining targeted enforcement with inclusive dialogue and rural economic support. Garba (2021) highlighted the cross-border dimension, demonstrating that banditry in the Nigeria-Niger borderlands cannot be contained through unilateral domestic operations, but requires deeper bilateral security engagement.

**Figure 2.**
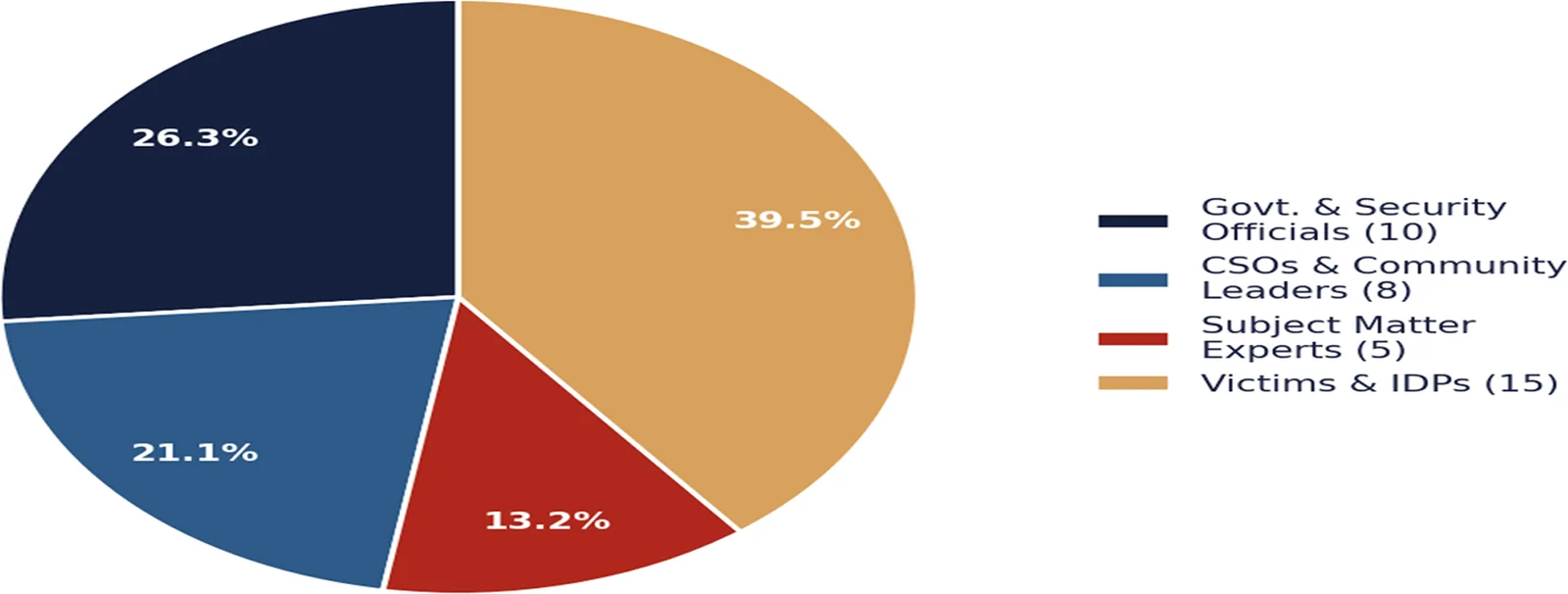
Sample distribution for interview. *Source:* Author’s Survey Data (2025).

A recurring theme across these studies is the absence of a coherent, integrated counter-banditry strategy. Irabor and Awofeso (2025) characterise government responses as reactive rather than strategic, undermined by poor coordination between federal and state authorities, vast ungoverned forest spaces, and weak border control. The literature on amnesty programmes is equally consistent: initial disarmament gains in Zamfara’s 2019 programme rapidly reversed due to broken government commitments, inadequate reintegration support, and the absence of transitional justice mechanisms (Sarkin Gulbi et al., 2024). Despite this substantial body of work, a critical gap remains in empirically grounded, multi-method assessments of combined federal and state response effectiveness specifically across the Northwest’s focal states; a gap this study addresses.

## 3. Theoretical framework

### 3.1 State fragility theory

This study is guided by three theoretical perspectives, combined not as parallel or competing lenses but as sequential, mutually reinforcing components of a single analytical architecture. State Fragility Theory supplies the diagnostic component, explaining why the governance vacuum enabling banditry emerges. The Human Security and SSR paradigm supplies the evaluative component, providing the standard against which government response is judged: not whether operations were conducted, but whether the security of ordinary people improved and was sustained. The Responsibility to Protect doctrine supplies the normative component, establishing the state’s obligation to protect its population and the criteria against which that obligation is assessed. Together, the three lenses move from cause, to evaluation, to obligation, and each generates a specific, testable proposition developed below.

Rotberg’s (2004) State Fragility Theory provides the foundational analytical lens for this study. Rotberg conceptualises state capacity along a spectrum from strong to failed states, arguing that states exist primarily to deliver political goods; security, rule of law, political participation, and basic services to their populations. When institutional capacity to deliver these goods deteriorates, governance vacuums emerge that non-state armed actors exploit to establish territorial control and parallel authority structures. Applied to Northwest Nigeria, the theory illuminates three interconnected failures: a security provision deficit (the inability to maintain a monopoly on legitimate violence in forested and border areas); an economic opportunity failure (chronic underdevelopment producing populations vulnerable to criminal recruitment); and judicial system weakness (near-zero prosecution rates generating pervasive impunity that reinforces bandit entrenchment). The theory predicts that without addressing these institutional dimensions, tactical security gains will remain inherently temporary. Call (2008) cautions against the totalising “failed state” label, arguing that it collapses analytically distinct deficits into a single category, and proposes instead that state failure be disaggregated into capacity, security and legitimacy gaps. This study adopts that narrower framing. The Nigerian state is not characterised here as failed; it is characterised as exhibiting a security gap, that is, a demonstrable shortfall in discharging the constitutional obligation under Section 14(2)(b) of the 1999 Constitution to make the security and welfare of the people the primary purpose of government, while continuing to function across other dimensions of statehood. This yields the study’s first proposition: tactical security operations unaccompanied by institutional rebuilding across the security, economic, and judicial dimensions will produce only temporally bounded reductions in violence (P1).

### 3.2 Human security and the SSR paradigm

The Organization for Economic Cooperation Development’s (OECD) Security Sector Reform framework (2007), grounded in human security principles, shifts evaluation criteria from state-centric military metrics to whether interventions improved the lived security of ordinary citizens. It identifies seven dimensions against which interventions should be assessed: effectiveness in protecting people; accountability to civilian oversight; affordability and sustainability; local ownership; transparency; adherence to the rule of law; and respect for human rights. This paradigm is particularly valuable for this study because it centres the analytical gaze on communities rather than states, asking not whether operations neutralised bandits but whether residents could farm safely, children could attend school, and women could access water without fear. It also foregrounds the importance of security sector reform as institutional transformation, and not merely military strengthening; encompassing police, judiciary, border management, and civilian oversight bodies. Following this paradigm, this study defines government response effectiveness as a multi-dimensional construct comprising three components: institutional coordination and consistency (the coherence and stability of inter-agency and inter-governmental collaboration measures); community trust and perceived adequacy (whether affected populations believe response efforts are genuine and sufficient); and participatory and restorative delivery (whether communities were consulted in intervention design and whether rebuilding and reintegration commitments were fulfilled). These components are operationalized through the six-item scale (Q13–Q18) analyzed in Section 5, which collectively distinguish effectiveness as a people-centered, outcome-oriented standard from mere activity or output (such as the number of operations conducted or personnel deployed). Applied to this study, the paradigm generates a second proposition: interventions characterized across these three dimensions will be perceived as more effective than interventions imposed without such engagement, irrespective of their tactical or kinetic success (P2).

### 3.3 Responsibility to protect

The Responsibility to Protect (R2P) doctrine, endorsed by the UN World Summit in 2005, provides a normative framework for evaluating state obligations toward populations facing systematic violence. Its three pillars including state responsibility to protect its own population (Pillar One); international responsibility to assist states lacking capacity (Pillar Two); and collective responsibility for timely and decisive response when states manifestly fail (Pillar Three), offer a graduated accountability framework. While R2P is conventionally applied to genocide and crimes against humanity, the Northwest Nigeria crisis which is characterised by systematic mass killings, sexual violence as a tool of terror, forced displacement, and attacks on both civilian and government infrastructures warrants analogous scrutiny. The doctrine’s emphasis on prevention, protection, and rebuilding provides evaluation criteria that extend beyond the kinetic, revealing Nigeria’s substantial deficits in post-conflict reconstruction, victim support, and accountability for atrocity crimes. This generates the study’s third proposition: government response measured against Pillar One obligations; prevention, protection, and rebuilding will show its most substantial deficits in the rebuilding and accountability components, since these are typically the last-prioritized phase of kinetic-led security responses (P3).

## 4. Methodology

### 4.1 Research design

This study adopts a mixed-methods sequential explanatory design, integrating quantitative analysis of survey data with qualitative thematic analysis of key informant interviews and comparative analysis of secondary security datasets. The pragmatist orientation prioritises methodological triangulation to strengthen internal validity. Quantitative analysis identifies patterns, trends, and statistical relationships; qualitative interpretation explains the mechanisms and contextual factors underlying those patterns; and comparative cross-state analysis contextualises findings within the variation of security experiences and policy responses across the Northwest.

### 4.2 Study area, sample, and participants

The Northwest geopolitical zone comprises seven states: Zamfara, Katsina, Kaduna, Sokoto, Kebbi, Kano, and Jigawa. This study is delimited to three of these states, purposively selected on four grounds. First, Zamfara, Katsina, and Kaduna consistently recorded the highest banditry incidence, fatality counts, and displacement figures across the 2017–2025 period, making them the empirical epicentre of the crisis. Second, each state pursued a distinct government response strategy. Zamfara relied primarily on military operations and an amnesty programme; Katsina emphasised negotiation, community policing, and cross-border engagement; and Kaduna adopted a hybrid approach combining intelligence-led operations with community vigilance providing the policy variation necessary for meaningful comparative analysis ( BSIL, 2025). Third, the three states span the geographic breadth of the Northwest, covering the western borderlands with Niger Republic (Zamfara), the northern border zone (Katsina), and the south-eastern corridor linking the Northwest to the Federal Capital Territory (Kaduna). Fourth, existing secondary data and institutional access were sufficient in these three states to support robust primary data collection, whereas the remaining four states Sokoto, Kebbi, Kano, and Jigawa while affected by banditry, have lower incident densities and less policy variation, offering diminishing analytical returns relative to the research questions. The three selected states therefore represent both the worst-affected and most analytically differentiated cases within the Northwest, justifying their selection as the primary unit of analysis.

A structured questionnaire was administered to respondents in the three states of Zamfara, Kaduna and Katsina, via Google Forms between February and May 2026. The total of 405 responses were received; 5 were excluded during data cleaning, 3 due to duplicate submissions and 2 due to systematic straight-line responses thereby yielding a final analytical sample of 400 valid responses. As Google Forms does not record the number of individuals who received the survey link, a traditional invitation-based response rate cannot be determined; the sample of 400 nonetheless meets the minimum derived from Taro Yamane’s (1967) formula applied to the study population. Required-field validation ensured item-level completeness across all substantive 24 Likert-scale items. The sample composition by stakeholder category was: Security/Government Officials (n = 110, 26.3%); Community Leaders (n = 85, 20.3%); Civil Society Organisations (n = 69, 16.5%); Victims and IDPs (n = 108, 25.8%); and others (n = 46, 11.0%). Gender distribution comprised 59.5% male and 40.5% female respondents; the dominant age bracket was 36 to 45 years (42.8%), reflecting the working-age population most directly affected by the crisis. Qualitative data were collected through thirty-eight semi-structured key informant interviews spanning all major stakeholder categories: security/government official, civil society organisation director, subject matter expert, internally displaced person, and traditional community leader.

### 4.3 Instruments and analysis

The questionnaire comprised 24 Likert-scale items (five-point scale: Strongly Disagree to Strongly Agree) organised around the five research objectives. Item validity was established through content validation by security studies experts and face validation by community gatekeepers. Quantitative analysis employed descriptive statistics (means, standard deviations, frequency distributions), one-sample t-tests (testing whether means differed from the neutral midpoint of 3.0), one-way ANOVA (testing inter-group differences across stakeholder categories), chi-square tests of association, and multiple linear regression (identifying predictors of perceived government effectiveness). Qualitative data were analysed using Braun and Clarke’s (2006) six-phase thematic analysis framework. Secondary quantitative data were drawn from Nigeria Watch, Reports on Violence in Nigeria (seventh to fifteenth editions), which provides national fatality counts disaggregated by cause and state on a calendar-year basis; from SBM Intelligence, The Economics of Nigeria’s Kidnap Industry (annual updates), which reports abduction and ransom figures nationally on a July–June cycle; from the National Bureau of Statistics, Crime Experience and Security Perception Survey 2024: and from Beacon Security and Innovations Limited (BSIL) Data Assessment Report: Government Response to Banditry, November 2025. These were analyzed through descriptive and time-series trend analysis. Because the sources use different reporting periods and units, they are treated as parallel series rather than as a single integrated dataset.

### 4.4 Ethical considerations

This study was conducted in accordance with the ethical guidelines of Nile University of Nigeria and the principles of the Declaration of Helsinki for research involving human participants. Nile University of Nigeria does not operate a formal institutional review board or research ethics committee. However, the study was designed and conducted in full accordance with the ethical principles of the Declaration of Helsinki for research involving human participants. Research ethics were carefully considered throughout the study design, encompassing voluntary participation, the right to withdraw without consequence, confidentiality and anonymity of all participants, and secure storage of all data collected. All participants were intimated on the study objectives, the voluntary nature of participation, and their right to withdraw at any time without consequence, and the confidentiality and anonymity protections in place. Informed consent was obtained from all participants before data collection commenced. For questionnaire respondents, consent was obtained electronically through a mandatory acknowledgement checkbox on the Google Forms survey platform, which participants were required to complete before accessing the survey items. For key informant interview participants, verbal informed consent was obtained prior to each interview. Written consent was not collected due to the sensitive security context of the study and the reluctance of participants to sign formal documents (particularly serving security officials and internally displaced persons). All data were stored securely, with access restricted to the research team, and no identifying information is reported in this article. Participants’ identities are protected through the use of stakeholder category codes rather than names or personally identifiable information.

## 5. Results and discussion

### 5.1 Quantitative trends in banditry (2017–2025)

Table 1 presents the longitudinal trends in fatalities, kidnapping and ransom values across the study period.

**Table 1.** Banditry fatalities, kidnapping-related fatalities, abductions and ransom values, Nigeria, 2017–2025.

Year	Banditry fatalities	Kidnapping-related fatalities	Reporting period	Abductions (incidents)	Ransom demanded	Ransom paid
2017	Not separately coded	290 (100 incidents)	No annual figure	—	—	—
2018	930	358 †	No annual figure	—	—	—
2019	2,067	115	No annual figure	—	—	—
2020	3,007	336	No annual figure	—	—	—
2021	4,407	575	No annual figure	—	—	—
2022	5,725	1,145	Jul 2021–Jun 2022	3,420 (500+)	₦6.531bn	₦653.7 m (≈$1.13 m)
2023	892	536	Jul 2022–Jun 2023	3,620 (582)	₦5.092bn	₦302 m
2024	1,452	425	Jul 2023–Jun 2024	7,568 (1,130)	₦10.995bn	₦1.048bn
2025	3,974	747	Jul 2024–Jun 2025	4,722 (997)	≈₦48bn	₦2.57bn (≈$1.66 m)

*Sources:* Banditry and kidnapping-related fatalities from Nigeria Watch, Reports on Violence in Nigeria, seventh to fifteenth editions (section 2.1.2 and section 2.1.3 of each), national totals by calendar year. Abduction and ransom figures from SBM Intelligence, The Economics of Nigeria’s Kidnap Industry, annual updates 2022–2025, national totals on a July–June cycle. The two series run on different reporting periods and are not directly comparable. Compiled by the authors.

† The Eighth Report gives 238 deaths in 76 incidents in its executive summary and 358 in section 2.1; the higher figure is used here.

The series below is national. It is presented to establish the macro-trend against which the three-state comparative analysis is set, since no published source reports fatalities, abductions and ransom values for the North West zone alone on a common annual basis across the study period. The national trend nonetheless establishes the temporal pattern against which state-level response outcomes in Section 5.2 are assessed.

The National Bureau of Statistics estimate of ₦2.2 trillion cited in the introduction derives from a household survey covering a single twelve-month period and is not comparable with the incident-counted totals reported above; the two are presented as separate measures rather than as a single series.

The longitudinal data reveal sustained escalation followed by sharp but unstable movement. Banditry fatalities rose from 930 in 2018, the first year in which banditry was coded as a distinct cause of lethal violence in Nigeria, to 5,725 in 2022, a more than six-fold increase, with the number of affected states expanding from 15 to nineteen and the Federal Capital Territory. Recorded fatalities then fell to 892 in 2023 and stood at 1,452 in 2024, before rising again to 3,974 in 2025 across sixteen states. The 2023 decline should not be read as straightforward evidence of operational success: Nigeria Watch attributes each fatality to a single dominant cause, so part of the movement reflects redistribution between the banditry, kidnapping and pastoral conflict categories rather than a reduction in violence as experienced. Kidnapping-related fatalities provide a partial check, peaking at 1,145 in 2022, falling to 536 and 425 in 2023 and 2024, and rising to 747 in 2025.

The kidnap economy shows nominal escalation without structural retreat. Abductions rose from 3,420 in 2021/22 to 7,568 in 2023/24 before falling to 4,722 in 2024/25, while recorded incidents moved from just over 500 to 1,130 and then 997. Ransom demanded rose from ₦6.531 billion to approximately ₦48 billion across four reporting years, and ransom confirmed paid from ₦653.7 million to ₦2.57 billion. In dollar terms, however, the movement is from roughly US$1.13 million to US$1.66 million, so the greater part of the apparent naira growth reflects currency devaluation rather than an expanding market, a point SBM Intelligence makes explicitly. The ratio of ransom paid to ransom demanded remained in single digits throughout, and the North West accounted for 2,938 of the 4,722 victims recorded in 2024/25, or 62.2 per cent.

A critical pattern across the data is the absence of a durable inflection point correlated with specific interventions. The 2019 Zamfara Amnesty Programme temporarily reduced attacks in 2020 before collapse and resurgence in 2021–2022. The November 2021 terrorism designation coincided with continued escalation rather than decline based on the events and numbers publically reported. Military operations produced measurable fatality reductions from 2023 onwards but could not prevent the 2025 school abductions in Kebbi and Niger states. This pattern is consistent with the theoretical prediction of State Fragility Theory: without institutional rebuilding alongside tactical operations, security gains are lost as soon as operational pressure relaxes.

### 5.2 Cross-State comparative analysis

Table 2 synthesises the banditry characteristics, government responses, and key outcomes across the five Northwest states and one Northcentral state. Niger state is included as an out of zone comparator: although outside the study’s sampling frame, its inclusion demonstrates the eastward spread of banditry beyond the Northwest and permits assessment of whether response patterns observed in the study states hold adjacent context. The comparison reveals a consistent pattern: responses anchored in integrated civil-military-community approaches achieved more durable, if still fragile, reductions in violence than exclusively kinetic strategies.

**Table 2.** Cross-state comparative analysis of banditry and government response (2017–2025).

State	Banditry characteristics	Government response	Key outcomes
Zamfara	Epicentre of early bandit activity, driven by illegal gold mining, cattle rustling and mass abductions. Ranks among the poorest states nationally, with a multidimensional poverty headcount of 78% ( National Bureau of Statistics, 2022)	Operation Hadarin Daji (2019–ongoing); periodic peace accords (2019, 2021); aerial strikes on forest hideouts. Amnesty Program; telecommunication shutdown	Temporary decline in 2020; resurgence by 2022 due to failed amnesty and inadequate reintegration. (BSIL)
Katsina	High-profile kidnappings including 2020 Kankara school abduction; cross-border bandit mobility.	Procurement of 43 APCs ( Sardauna, 2025); joint military task force; community cooperation programme; peace negotiations with bandit commanders. State level amnesty programme	Continuous attacks despite security; Highway attacks stabilised but rural raids persisted. Amnesty/peace deals failed to achieve lasting peace as bandits migrate from one area to another, coupled with unfulfilled development promises ( BSIL, 2025).
Kaduna	Urban-rural divide in insecurity; attacks along Abuja-Kaduna highway; Birnin Gwari forest as bandit sanctuary.	Operation Thunder Strike and Whirl Punch; establishment of Kaduna Vigilance Service (2023); community-military patrols. Reintegration of victim attempts.	Highway attacks fell in 2024; rural raids continued; limited community trust. (interviewee)
Sokoto	Smaller but intensifying attacks along Illela-Gwadabawa axis; cross-border links with Niger Republic.	Vigilante integration and dialogue with border communities (2023); federal troop reinforcement.	Bandit networks restructured under new leadership; kidnapping-for-ransom remains frequent.
Kebbi	Cross-border spillover from Zamfara; Danko-Wasagu and Ngaski LGAs worst affected; 2025 school abductions.	Ariel raids and land patrol to protect indigenes. Occasional Distribution of relief materials to victims.	Improved local vigilance but frequent retaliation attacks.
Niger	Banditry expanded eastward; Shiroro and Rafi LGAs severely affected; 2025 school abductions.	Federal troop surge under Operation Whirl Stroke and Operation Sahel Sanity; improved community liaison structures.	Drop in reported kidnappings by mid-2025, subsequent reversal following November 2025 school abductions.

The data reveal that Zamfara, despite the most intensive military engagement, recorded the most pronounced implementation failures in amnesty programming. Kaduna’s hybrid model which combinine intelligence-led operations with community vigilance and targeted reintegration protocol produced more sustained highway security improvements, though rural areas remained exposed. Katsina states’ peace dialogue has continued to raise concerns among residents as activities of bandits shift from areas with peace deals to areas without. Niger State’s relative improvement from coordinated federal troop deployment and community liaison structures; its subsequent reversal following November 2025 school abductions, illustrates both the promise and fragility of gains built on operational presence without structural governance investment.

### 5.3 Primary survey findings

Table 3 summarises the composite statistical outcomes across the four research objectives addressed through the survey instrument.

**Table 3.** Composite statistical results by research objective.

Research Objective	Items	Mean	SD	t-stat	p-value	Decision
Impact on national security (Obj.1)	Q1–Q5	4.31	0.77	44.035	< 0.001	Reject H _0_ — significant impact confirmed
Government response measures (Obj.2)	Q6–Q12	3.21	—	7.891	< 0.001	Above neutral; root causes not addressed (Q7 mean = 2.01)
Effectiveness in protecting lives/property (Obj.3)	Q13–Q18	2.61	—	−13.291	< 0.001	Below neutral — government perceived as ineffective
Challenges to effectiveness (Obj.4)	Q19–Q24	2.54	—	−15.334	< 0.001	Below neutral — trust & coordination as primary challenges

Research Objective 1 findings were clear and consistent: across all five dimensions measuring banditry’s impact on national security, respondents recorded a composite agreement of 92.4% (composite mean = 4.31, SD = 0.77, t = 44.035, p < 0.001). The item recording highest agreement was the direct impact on the lives of settlers and indigenes (mean = 4.42, SD = 0.75, 93.3% agreement), consistent with the documented humanitarian toll. The near-unanimous perception that bandits exercise greater territorial control than government in some areas (mean = 4.29, SD = 0.74, 94.1% agreement) directly corroborates Rotberg’s (2004) state fragility characterisation.

Research Objective 2 results tell a more complex story. The composite mean of 3.211 reflects moderate acknowledgement that government intervened, with 61.3% agreeing government played a role (mean = 3.52). However, the most striking finding is the near-consensus rejection of the proposition that government addressed root causes of banditry: 76.1% of respondents disagreed (mean = 2.01, t = −19.657, p < 0.001), representing the lowest mean and most statistically significant negative finding across the entire instrument. A related finding carries equal weight: 59.7% agreed that amnesty efforts emboldened criminality rather than rehabilitating bandits (mean = 3.60, t = 13.160, p < 0.001) a finding with direct policy implications for the design of any future peace engagement.

Research Objective 3 produces the sharpest verdict on government performance in the dataset. The composite mean of 2.614, this is well below the neutral midpoint (t = −13.291, p < 0.001). This indicates that respondents across all categories perceived government as ineffective in protecting lives and property. The most severe finding was community trust in government-led security efforts: only 23.8% of respondents agreed, while 76.5% disagreed (mean = 2.127, t = −16.289, p < 0.001). A one-way ANOVA revealed no statistically significant difference in these perceptions across respondent categories; civilian, victim, community leader, civil society, or security official (F(4, 400) = 0.849, p = 0.495). This confirms that the perception of government ineffectiveness is structural rather than group-specific.

Research Objective 4 identified six structural challenges to effectiveness (composite mean = 2.541, t = −15.334, p < 0.001). Ranked by severity: erosion of community trust (mean = 2.127); perception that government is not doing enough (mean = 2.231); absence of community consultation before interventions (mean = 2.303); exclusion of civilian perspectives from policy design (mean = 2.543); lack of effectiveness measurement systems (mean = 2.667); and frequent policy changes (mean = 3.362, significantly above neutral). The chi-square test confirmed that these challenge perceptions are shared broadly across all respondent categories (χ ^2^(4) = 8.569, p = 0.073), indicating structural rather than group-specific problems.

### 5.4 Predictors of security effectiveness: regression analysis

Table 4 presents the full regression model output, including standardised coefficients, t-values, and significance levels for each predictor.

**Table 4.** Multiple linear regression — predictors of perceived government effectiveness. Model: Perceived Effectiveness ~ Trust + Consultation + Rebuild + Collaboration + Frameworks + Amnesty + Negotiations. R ^2^ = 0.895|Adjusted R ^2^ = 0.893|F(7, 380) = 461.4, p < 0.001.

Predictor variable	Coefficient (β)	t-value	p-value	Significance
Community Trust	β = 0.247	t = 22.552	p < 0.001	Strongest predictor ***
Community Consultation	β = 0.194	t = 16.123	p < 0.001	Second strongest ***
Rebuilding Communities	β = 0.187	t = 16.180	p < 0.001	Highly significant ***
Inter-govt Collaboration	β = 0.165	t = 17.782	p < 0.001	Highly significant ***
Protection Frameworks	β = 0.028	t = 2.187	p = 0.029	Marginally significant *
Amnesty/Reintegration	β = 0.015	t = 1.271	p = 0.204	Not significant
Negotiations/Dialogue	β = −0.005	t = −0.392	p = 0.695	Not significant

*** p < 0.001;

* p < 0.05; NS = Not Significant. Source: Authors’ primary data.

The regression model is exceptionally strong, explaining 89.5% of variance in perceived security effectiveness (R ^2^ = 0.895). Four predictors are statistically significant at the highest threshold (p < 0.001). Community trust is the most powerful predictor (β = 0.247), followed by community consultation (β = 0.194), programmes to rebuild and reintegrate displaced communities (β = 0.187), and inter-governmental collaboration (β = 0.165). A fifth predictor, protection frameworks, achieves marginal significance (β = 0.028, p = 0.029). Two frequently deployed intervention types; amnesty/reintegration and negotiations/dialogue, are not statistically significant predictors (p = 0.204 and p = 0.695, respectively). This finding does not dismiss these tools conceptually but establishes that they generate no measurable effectiveness gains in the absence of the foundational conditions of trust, consultation, and coordination.

The regression findings carry significant strategic implications. Government has invested disproportionately in military operations, amnesty programmes, and diplomatic negotiations, precisely the tools that the data indicate are least predictive of effectiveness. The four variables that most strongly predict effectiveness — trust, consultation, rebuilding, and coordination have received the least systematic investment. This inversion of the resource-evidence relationship is the core governance failure the study identifies.

### 5.5 Qualitative thematic analysis

Five strategic themes emerged from cross-analysis of key informant interviews, directly corroborating and elaborating the quantitative findings.

**Theme 1 — Tactical activism without strategic coherence:** All five interview stakeholder categories across their divergent institutional positions, characterised government response as exhibiting fragmentation rather than integration. A security official acknowledged that simultaneous federal clearance operations and state amnesty negotiations in Zamfara in 2020 created “mixed messaging that bandits exploited.” A subject matter expert described government response as “disconnected initiatives rather than elements of a unified strategy,” while a community leader observed that military operations “produced short-term results but bandits simply relocated or waited out operations.” That this critique is shared across institutional lines is analytically significant: the critique of strategic incoherence is not confined to civilian observers but is shared by those implementing security policy itself.

**Theme 2 — Structural imbalance between kinetic and developmental investment:** All civilian participants and security officials alike emphasised the inadequacy of military-dominant responses. A subject matter expert estimated that “80% or more goes to kinetic operations, perhaps 20% to governance and development — this should reverse.” A CSO leader stated that “development prevents recruitment more effectively than bullets.” A security official acknowledged: “without rapid deployment of governance, development, and policing to fill vacuums, bandits simply return” — directly explaining the whack-a-mole pattern confirmed by the quantitative trend data.

**Theme 3 — Erosion of community trust as the operative constraint:** The erosion of trust — the strongest statistical predictor (β = 0.247) — was unanimously corroborated by all respondent categories. Only 32% of high-conflict LGA respondents felt safe during daytime; only 8% felt safe at night. A CSO leader stated that government “makes decisions in Katsina town or Abuja without asking what communities actually need.” A community member described the marginalisation of traditional institutions. An internally displaced person articulated the consequence starkly: “We feel forgotten; useful during elections for votes, abandoned otherwise.” The security official’s silence on community consultation in describing operational planning suggests that participatory security design is absent from military doctrine.

**Theme 4 — Implementation collapse of amnesty and DDR:** All participants acknowledged the conceptual potential of amnesty while unanimously condemning its execution. Zamfara’s 2019 programme was described as “transactional — cash for guns — without transforming underlying conditions” (CSO). A subject matter expert drew an instructive comparison to Colombia, where “successful DDR required multi-year commitment spanning years, not months.” A community leader who served on the negotiating committee confirmed that “government’s failure to fulfil promises undermined the process: stipends delayed, vocational training never materialised.” The Katsina model of selective, monitored reintegration of specific commanders represents the only example of localised success, conditional on sustained implementation.

**Theme 5 — Formalisation of community defence as strategic necessity:** The reliance on Yan Sakai and similar vigilante formations was characterised across all respondent categories as a symptom of state incapacity rather than deliberate strategy. A subject matter expert criticised “lack of oversight, training, and accountability mechanisms” which created ethnic targeting and extrajudicial conduct. A traditional leader proposed that “government should establish a Peace-Building Fund accessible to traditional councils” to anchor community security within accountable governance frameworks.

## 6. Discussion

### 6.1 Theoretical linkages

The empirical findings align closely with all three theoretical paradigms. P1 (State Fragility Theory) is supported; tactical security operations without institutional rebuilding produce temporally bounded gains, consistent with Rotberg’s (2004) prediction. The economic dimension of this failure is evident in the study states, where Zamfara and Katsina rank among the poorest nationally on the multidimensional poverty headcount ( NBS, 2022) while sustaining the heaviest banditry burden. Respondents’ own assessments corroborate the mechanism; community rebuilding emerged as the third-strongest predictor of perceived government effectiveness (β = 0.187, p < 0.001), indicating that populations experiencing tactical operations without accompanying development investment do not perceive durable security gains. The near-total absence of effective banditry prosecutions in line with established laws, embodies the judicial system weakness Rotberg identifies as constitutive of state fragility. Also the bandits’ exercise of parallel governance confirmed at 94.1% agreement by survey respondents evidences the security gap Call (2008) identifies, in which the state’s monopoly on the provision of protection is displaced by non-state actors within defined territory, without the wider collapse of statehood the failed-state designation implies.

P2 (The Human Security and SSR Paradigm) is supported. Evaluated against the framework’s seven dimensions, government responses consistently fail the people-centred standard: only 32% of community members felt safe during daytime; no compensation or property recovery mechanisms existed for victims; rehabilitation programmes reached less than 1% of those requiring support. The framework’s emphasis on community ownership directly corroborates the regression finding that consultation (β = 0.194) is the second strongest predictor of effectiveness. The pattern of top-down operations without community input confirmed by 64.9% of respondents who disagreed that communities were consulted before interventions, represents precisely the accountability and ownership deficits that SSR theory predicts will undermine effectiveness.

P3 (R2P) is likewise supported, revealing the normative dimension of these failures. Nigeria’s Pillar One obligations; prevention, protection, and rebuilding, were inadequately fulfilled across all three phases. Prevention was neglected as government invested overwhelmingly in reactive military operations rather than addressing land conflicts, youth unemployment, and arms proliferation. Protection was geographically selective, creating “security islands” in urban centres while leaving vast rural populations exposed. Rebuilding was essentially absent: little to no compensation funds, no psychosocial support, or transitional justice mechanisms. The international community’s limited engagement with the Northwest crisis is still limited despite the systematic scale of violence. This therefore raises questions about Pillar Two fulfilment that warrant further scholarly attention.

### 6.2 The strategic paradox of nigerian counter-banditry

The study’s most significant contribution is the identification of a systematic inversion between resource allocation and evidential effectiveness. The regression model establishes that community trust, consultation, rebuilding, and inter-governmental collaboration are the four most powerful predictors of security effectiveness. Yet government has consistently concentrated its resources in the two tool types that the data establish as least predictive of effectiveness, that is, kinetic operations and amnesty programmes designed without the foundational conditions of trust and consultation. This is not a failure of intent but of institutional architecture: Nigeria possesses military capacity to clear territory but seems to lack the governance capacity, resource allocation discipline, and sustained political commitment to hold and build in the aftermath.

This finding resonates with and extends the existing literature. Aina et al. (2023) documented the limits of shock-and-awe tactics without governance integration; this study quantifies those limits with statistical precision and adds the crucial finding that community trust is the operative mechanism through which governance investments translate into security outcomes. Fallata et al. (2024) identified reactive fragmentation as a core problem; this study establishes through regression analysis that the antidote is not a different type of reactive intervention but a foundational reorientation toward trust-building, participatory design, and inter-governmental coordination.

## 7. Recommendations

Based on the integrated findings, five recommendations grounded in the study’s empirical findings are advanced for policymakers, security agencies, and development actors.

**First, adopt a unified Northwest Security Grand Strategy.** The absence of a coherent, multi-year/level counter-banditry strategy confirmed across all respondent categories demands immediate remedy. Federal and state governments should jointly develop an integrated strategy articulating a clear theory of victory, success metrics, and timelines, with a unified Northwest Security Command integrating military, police, intelligence, and civilian agencies under shared coordination. The strategy must mandate the “clear-hold-build” doctrine: military clears areas; sustained police presence holds them; civilian agencies build through services and development.

**Second, rebalance resource allocation from kinetic to governance and development.** The current estimated 80:20 ratio of kinetic to governance spending must be reviewed toward a 40:60 target within three years. For every cleared area, Civilian Governance Rapid Deployment teams comprising local government officials, healthcare workers, and agricultural extension officers should be mobilised within 30 days. A Civilian Protection Protocol should be institutionalised, establishing rules of engagement protecting civilian objects, mandatory community notification before operations, and grievance mechanisms for communities affected by security force conduct.

**Third, redesign DDR programmes to international standards.** Any future amnesty engagement must be anchored in a DDR programme meeting international benchmarks: verified weapons surrender; a minimum 18-month reintegration pathway including psychosocial support, vocational training, and business startup capital; independent civil society monitoring; ring-fenced and corruption-proof funding; and regular public outcome reporting. A Northwest Transitional Justice Commission should be established to administer victim compensation, truth-telling forums, and community reconciliation, drawing on adapted West African precedents such as Sierra Leone’s TRC model.

**Fourth, institutionalise community participation as a mandatory planning requirement.** Given that community trust (β = 0.247) is the strongest predictor of security effectiveness, it must be treated as a strategic input rather than an optional consultation. Community Security Committees should be established in all affected Local Government Areas with formal advisory roles in security planning and evaluation. A Northwest Peace-Building Fund should resource traditional institutions (Emirs, District Heads, Sarkis) for certified conflict mediation and community reconciliation activities, with accompanying security protection for traditional leaders engaged in sensitive peace work.

**Fifth, address structural drivers through targeted long-term development investment.** The concentration of banditry in states with the highest multi-dimensional poverty demands multi-year government commitment to Northwest-targeted development. Priority investments should address youth unemployment through public works, apprenticeship schemes, and skills development; universal primary and secondary education access to reverse the documented 19–27% dropout rates; agricultural rehabilitation including land restoration, input subsidies, and market infrastructure; and a Victims’ Relief and Recovery Fund to provide standardised compensation for losses of life, livestock, and property. These investments must be accompanied by transparent, independently audited resource management to prevent the corruption and diversion that may have undermined past programmes.

## 8. Conclusion

This study has provided an empirically grounded assessment of Nigerian government responses to armed banditry in Northwest Nigeria between 2017 and 2025. Drawing on survey data from 400 respondents, thirty-eight key informant interviews, cross-state comparative analysis, and a nine-year longitudinal dataset of security indicators, the findings point consistently toward the same verdict: government responses have been tactically active but strategically incoherent, achieving temporary violence suppression without sustainable civilian protection or structural transformation.

Three conclusions carry particular weight. First, banditry constitutes a multidimensional national security crisis and not merely a regional crime problem which was confirmed at overwhelming statistical significance across all measured dimensions. Second, the perception of government ineffectiveness is systemic, shared across all respondent categories including security and government officials themselves, a finding that speaks to structural institutional failure rather than individual incompetence. Third, and most importantly, the study has empirically identified what works: community trust, participatory security design, rebuilding investment, and inter-governmental coordination explain 89.5% of the variance in perceived effectiveness. The tools governments have prioritized (kinetic operations and sometimes stand-alone amnesty programmes) are, by contrast, statistically insignificant predictors of effectiveness in the absence of these foundational conditions.

The path to sustainable security in the Northwest is empirically mapped. It requires a fundamental reorientation from reactive militarization to proactive governance; from state-centric threat neutralization to people-centered protection; from episodic crisis management to sustained institutional commitment. Nigeria has the strategic knowledge and institutional foundations to make this shift. What is required is the resource discipline, and sustained commitment to translate evidence into governance transformation. The millions of Nigerians living under the constant threat of banditry in the Northwest cannot afford a further decade of tactical activism without strategic change.

## Data Availability

The datasets supporting the findings of this study is made available in an open-access repository. The primary survey data, including anonymised questionnaire responses (n = 400) and all associated variables (respondent category, state, gender, age group, educational attainment, and all 24 Likert-scale item responses), is deposited in the Figshare open-access repository. The dataset is published under a Creative Commons Attribution 4.0 International (CC BY 4.0) licence, permitting unrestricted reuse with appropriate attribution. The dataset is available at: https://doi.org/10.6084/m9.figshare.32688162 ( John Zinna & Garba, 2026). The dataset includes: all individual item-level Likert-scale responses across 24 survey items from 400 respondents, underpinning the reported means, standard deviations, and frequency distributions; the values used in all statistical analyses including the one-sample t-tests, one-way Analysis of Variance (ANOVA), chi-square tests, and multiple linear regression model; and demographic variables including respondent category, state of residence, gender, age bracket, educational qualification, marital status, and occupation. Secondary data sources cited in this article Nigeria Watch, BSIL, NBS (2024), SBM Intelligence (2025) are publicly available from their respective organizations as cited in the References section. Raw interview transcripts cannot be shared in full due to participant confidentiality obligations; access may be requested from the corresponding author subject to ethics conditions. The extended data — including the survey questionnaire instrument and the key informant interview guide in two versions, one for CSOs, Victims and Community Leaders, and one for Subject Matter Experts and Security/Government Officials — have been uploaded to the repository alongside the primary dataset. A participant consent form template is not included in the repository as verbal informed consent was obtained from interview participants rather than written consent.
