# Supplementary material for: Assessing the Impact of Nigerian Government Responses to Armed Banditry in Northwest Nigeria, 2017–2025
Source: Open Res Afr. 2026 Oct 5;9:43. Originally published 2026 Jul 8. [Version 2] doi: 10.12688/openresafrica.16703.2 (PMC13714415; doi:10.12688/openresafrica.16703.2)
Supplement: Peer review history

# Reviewer report: Reviewer response for version 2

**DOI:** 10.21956/openresafrica.18116.r36657

**Published:** 2026-10-27

**Authors:** Tosin Osasona

**Affiliations:** 1 Centre for Public Policy Alternatives, Lagos, Nigeria

The  review addressed all issues raised.

Is the work clearly and accurately presented and does it cite the current literature?

Yes

If applicable, is the statistical analysis and its interpretation appropriate?

I cannot comment. A qualified statistician is required.

Are all the source data underlying the results available to ensure full reproducibility?

Partly

Is the study design appropriate and is the work technically sound?

Partly

Are the conclusions drawn adequately supported by the results?

Partly

Are sufficient details of methods and analysis provided to allow replication by others?

Partly

Reviewer Expertise:

Organized criminal groups in the African Sahel; Conflcit analysis; international humanitarian law

I confirm that I have read this submission and believe that I have an appropriate level of expertise to confirm that it is of an acceptable scientific standard.

# Reviewer report: Reviewer response for version 2

**DOI:** 10.21956/openresafrica.18116.r36691

**Published:** 2026-10-15

**Authors:** Victor Chukwugekwu Ebonine

**Affiliations:** 1 University of Benin, Benin City, Edo, Nigeria

**Comments**

 I am particularly thrilled when I saw the title of the paper because Nigeria is in need of scholarship that can provide useful recommendations to tackle raging banditry across the studied states and elsewhere in Nigeria. The authors used mixed methods, including time-series data, to draw inferences as to the oxygenation and implications of banditry in Zamfara, Kaduna and Katsina. However, the paper suffers from major theoretical, methodological and analytical deficiencies. I review according to each heading or section of the paper.

**Objective:**

 I noticed a sort of broad objective: **“** **This study assesses the impact of Nigerian government responses to banditry during this period, examining whether interventions measurably reduced violence, strengthened state authority, restored human security, and addressed structural drivers of conflict.”** Whether this is what the authors identified as five objectives is confusing. As it is, there is a need for clearer delineation of the objective or objectives of the study.  

**Research questions:**

 The authors stated: “ **The research poses four core questions: What major policies and strategies did government deploy to combat banditry in the Northwest between 2017 and 2025? To what extent did these responses reduce violence, improve security, and restore socio-economic stability? How did affected communities and stakeholders perceive and experience government interventions? And what lessons can be drawn to inform more sustainable and effective counter-banditry approaches? **Assuming the above objective or objectives were taken, these research questions do not align with them. Each research question should correspond to each research objective. Here, the authors state that they have five objectives and four research questions. That is confusing. Moreover, I would like both research objectives and research questions to be clearly stated as separate headings.

**Research propositions:**

 I am confused as to how the authors derived their propositions mainly from theoretical frameworks without corresponding literature interrogation. There should have been a heading for empirical review under the literature review for each of the objectives highlighted. This would have allowed for clear propositions or hypotheses. The three propositions should flow from both empirical literature and theoretical standpoints. The literature review section is scanty, uncritical, and highly descriptive.

**Literature Review:**

 The literature review section is highly uncritical and journalistic. More literature should be interrogated to bring out gaps. Possibly, an empirical review section should be provided. Under this section, the paper needs robust copyediting and proofreading. The prevalence of grammatical, punctuation and sentence errors across the work vitiates readability, comprehension and logical flow. For instance, the last paragraph under the **evolution of banditry in northwest Nigeria **is clumsy and affects readability. For instance, it partly reads: “The following structural drivers have been consistently identified in the literature; Persistent poverty and youth unemployment, with the Northwest recording poverty rates exceeding 70% and youth unemployment surpassing 45% between 2017 and 2024, as stated by the National Security Adviser – Nuhu Ribadu in 2024 (Maruf, 2024); this has created conditions of material desperation that criminal networks exploit. Another Driver is that environmental degradation and climate-induced displacement have intensified farmer-herder competition…” I suggest the authors break it down and correct all grammatical and sentence errors.

 Similarly, under **evaluations of government response, **the part that reads: “Aina et al. (2023) evaluated Nigeria’s military “shock and awe” strategy and found that while deployments temporarily degrade bandit mobility, fragmentation, terrain familiarity, and criminal adaptation blunt deterrence effects without integrated governance measures, Fallata et al. (2024) found government actions to be reactive and fragmented, with limited community engagement and weak rural policing undermining intelligence capacity.” Is incomprehensible.

**Theoretical Framework**

 The paper used three reinforcing theories: State fragility, Human Security and the SSR Framework and R2P. This is plausible and merits commendation. However, they lacked empirical linkage and analytical strength. The authors said that State fragility is diagnostic. Throughout the work, I find it difficult to capture the variables that emanated from the diagnosis. The same applies to other theories deployed. How does each contribute distinct analytical insights in the study?

  The authors adopted the first pillar of R2P with the following assumptions: prevention, protection, and rebuilding. Yet, there were no empirical tests for these adopted assumptions. There was no operational definition of terms to enable measurement. Moreover, I doubt the place of R2P in the study because it is a variant of the human security framework. Therefore, I suppose the Human Security Paradigm can cover it.

 As I stated earlier, the propositions that emerged from the theories lacked empirical bases. The authors should address this.

**Methodology**

 The authors state that **this study adopts a mixed-methods sequential explanatory design **without justification. Why did they adopt this design over and above all other designs? How does it suit the present study? Are there previous related studies that adopted it? How did it help their studies? These are critical questions to address under research design.

 I like the authors’ justification for selecting the three studied areas. However, they did not justify why they chose 2017 to 2025. Additionally, under the **Study area, sample, and participants, **the authors muddled the explanation with the participants’ demographic analysis. This should be separated for clarity.

 The study did not provide a causal-identification strategy for evaluating whether the government responses were solely responsible for events. For instance, a decline in recorded fatalities after a military operation does not necessarily mean that the operation caused the decline. Other factors, including changes in reporting, the movement of armed groups, changes in classification or simultaneous interventions, could contribute.

**Population/Sample Size and Sampling technique**

 I saw the use of Taro Yamane (1967) for sample size calculation, and I wondered where the population was derived from. Throughout the work, I could not find the population of the various critical stakeholders selected for the study from which a sample was chosen. When selecting the respondents, the authors need to clarify these issues.

- How were participants recruited and selected?
- What procedures ensured that the sample adequately represented the different categories of stakeholders?
- How were respondents in insecure or displaced communities reached?
- Were people without reliable internet access systematically excluded?
- What population was used to justify the sample size of 400?

The authors indeed included diverse critical stakeholders in the study. However, they did not amount to a representative sample. The use of an online questionnaire might have excluded those in the remotest areas with limited internet infrastructure.

**Instruments and analysis**

 The authors note that **Item validity was established through content validation by security studies experts and face validation by community gatekeepers. **This is confusing. Who are the security experts? Where are they located? How were the communities’ gatekeepers located: online or in person?

 I think that there are too many statistical techniques here: chi-square, t-test, ANOVA, regression and descriptive techniques. I think some are overlapping. For instance, a t-test can do what ANOVA can do. Why the two together?

 Secondary quantitative data from different enumerated sources were used as shown in Table 1. However, I suppose that the data should be for the three selected study areas rather than the whole of Nigeria. If it is meant to cover kidnapping-related events across Nigeria, this should be merged with a relevant heading under the literature review rather than creating a separate analytical heading for it. Moreover, a line graph should be used to denote the trend to ease understanding. Regardless, the table ought to be for the three selected states only, with trend analysis.  

**Ethical Considerations**

 The paper reads: **This study was conducted in accordance with the ethical guidelines of Nile University of Nigeria and the principles of the Declaration of Helsinki for research involving human participants. Nile University of Nigeria does not operate a formal institutional review board or research ethics committee. **So, how did the authors develop the university’s ethical guidelines used for the study without an ethics board? Who satisfied the guidelines before use?

 Additionally, there is a need for clarity between participants and respondents. From the paper, I can understand that those who participated in the interviews were also part of those surveyed with a questionnaire. Meanwhile, the paper states that **written consent was not collected due to the sensitive security context of the study and the reluctance of participants to sign formal documents (particularly among security officials and internally displaced persons. **So, how is it possible to obtain informed consent from individual respondents but not possible with the same individuals as interviewees?  

**Results and discussion**

 I think there are inconsistencies with the results. First, the paper reports an ANOVA result of:

 F (4,400)=0.849,p=0.49.

- Between groups: 5−1=45-1=4
- Within groups: 400−5=395400-5=395

The expected result would therefore be reported as F(4,395), assuming there were no additional complications. The reported denominator degrees of freedom of 400 do not match that calculation.

 Also, the degrees of freedom in the regression table (F(7,380)=461.4,p<0.001) are questionable. To calculate the degrees of freedom: df = =N−k−1

 Where:

- N = number of observations used in the regression.
- k = number of predictors.
- 11 = the intercept, also called the constant.

For the article, assuming all 400 observations were used: 400−7−1=392​

 Further, the t-test result is also suspicious and needs clarification. For instance, the authors **compare composite means with a neutral midpoint of 3.0 on a five-point Likert scale. **This suggests that a composite mean below 3.00 is expected to produce a negative t-statistic **. **For instance, authors **r**eport a composite mean of 2.614 for perceived government effectiveness and a positive t-statistic of 13.291. They also report a mean of 2.127 for community trust alongside a positive t-statistic of 16.289. This is confusing. There should have been a negative t-statistic.

 The authors also interpreted the chi-square result wrongly. The paper reports that (χ2(4)=8.569,p=0.073) confirms that perceptions of government effectiveness are shared broadly across respondent categories.  When considered against a 5% significance level, the value (0.073) is not statistically significant.

 I also think that the regression result R ^2^ (R2=0.895, Adjusted R2=0.893) is unusually high for a cross-sectional survey involving perceptions of complex government interventions. This should be properly cross-checked.

**Thematic analysis**

 The themes were not sufficiently developed with participants’ direct words. Moreover,  theme three reads: **The erosion of trust — the strongest statistical predictor (β = 0.247) — was unanimously corroborated by all respondent categories. Only 32% of high-conflict LGA respondents felt safe during daytime; only 8% felt safe at night.**  What is statistics doing in a qualitative report?

 The section on theoretical linkages and discussion of findings needs robust engagement with the literature. How do the quantitative and qualitative results complement or contrast each other?  A section dedicated to data synthesis may suffice here.

**Recommendations**

 Very commendable that recommendations flow from findings. However, they require measurable indicators and clear lines of responsibility.

**Keywords:**

 No identifiable keywords in the paper.

Is the work clearly and accurately presented and does it cite the current literature?

No

If applicable, is the statistical analysis and its interpretation appropriate?

No

Are all the source data underlying the results available to ensure full reproducibility?

Partly

Is the study design appropriate and is the work technically sound?

No

Are the conclusions drawn adequately supported by the results?

Partly

Are sufficient details of methods and analysis provided to allow replication by others?

Partly

Reviewer Expertise:

security studies, political economy and development studies

I confirm that I have read this submission and believe that I have an appropriate level of expertise to confirm that it is of an acceptable scientific standard, however I have significant reservations, as outlined above.

# Reviewer report: Reviewer response for version 1

**DOI:** 10.21956/openresafrica.17896.r35270

**Published:** 2026-07-24

**Authors:** Paul Akere Irabor

**Affiliations:** 1 Olabisi Onabanjo University, Ago-Iwoye, Ogun State, Nigeria

The topic of the article is appropriate for the state of banditry in Northern Nigeria. The objectives of the article are clear and have been achieved. The methodology (mixed method) is appropriate and leads to the achievement of the objectives.

  However, the literature review is somewhat defective. The researcher needs to cite authors appropriately. For instance, Irabor and Awofeso (2025) were wrongly cited as: Irabor P, Awofeso O: Intelligence-led policing and the fight against banditry in Kaduna. Nigerian Journal of Social Sciences. 2025; 18(1): 55–74.

 The correct citation for Irabor and Awofeso (2025) is: Irabor, P.A. and Awofeso, O. (2025). Analysis of Government Response to Curb Banditry in Nigeria. *Journal of Social Studies. 31, 6 *DOI:https://doi.org/10.20428/jss.v31i6.2989.

 Several other citations are also not correct. The researcher needs to go over the references and cite authors correctly.

Is the work clearly and accurately presented and does it cite the current literature?

Partly

If applicable, is the statistical analysis and its interpretation appropriate?

Yes

Are all the source data underlying the results available to ensure full reproducibility?

Yes

Is the study design appropriate and is the work technically sound?

Yes

Are the conclusions drawn adequately supported by the results?

Yes

Are sufficient details of methods and analysis provided to allow replication by others?

Yes

Reviewer Expertise:

Security and Development Studies, and Comparative Politics.

I confirm that I have read this submission and believe that I have an appropriate level of expertise to confirm that it is of an acceptable scientific standard, however I have significant reservations, as outlined above.

# Response

**Published:** 2026-08-31

**Authors:** Aitse John Zinna

**Affiliations:** Political Science and International Relations, Nile University of Nigeria, Abuja, Federal Capital Territory, Nigeria

We thank you for the affirmative assessment of the topic's relevance, the clarity of the objectives, the appropriateness of the mixed-methods design and your observations. The Irabor and Awofeso entry now reads: Irabor PA, Awofeso O: Analysis of government response to curb banditry in Nigeria. Journal of Social Studies. 2025; 31(6). https://doi.org/10.20428/jss.v31i6.2989. Both the in-text citation and the reference list have been amended. We have gone through the reference list in full, verifying every entry against publisher or repository records. Entries no longer cited in the revised manuscript have been removed. We are grateful for these observations, which have improved the manuscript.

# Reviewer report: Reviewer response for version 1

**DOI:** 10.21956/openresafrica.17896.r35269

**Published:** 2026-07-14

**Authors:** Tosin Osasona

**Affiliations:** 1 Centre for Public Policy Alternatives, Lagos, Nigeria

The study addresses one of the leading causes of Nigeria's  humanitarian crisis  and makes a valuable contribution to the scholarship on armed banditry through its mixed-methods approach, which is a welcome departure from the predominantly qualitative literature.

 However, the theoretical framework is relatively weak. The combination of theories lacks coherence and does not provide a strong analytical foundation for the study. Moreover, the theories are applied descriptively rather than being used to generate hypotheses or guide the analysis.

 The study also lacks conceptual clarity regarding its key variable, which is government effectiveness. The manuscript does not clearly define what "effectiveness" entails or the specific dimensions against which state responses are being evaluated.

 Overall, the study advances the scholarship on banditry in Nigeria, but strengthening its theoretical grounding and conceptual clarity would significantly enhance its contribution.

Is the work clearly and accurately presented and does it cite the current literature?

Yes

If applicable, is the statistical analysis and its interpretation appropriate?

I cannot comment. A qualified statistician is required.

Are all the source data underlying the results available to ensure full reproducibility?

Partly

Is the study design appropriate and is the work technically sound?

Partly

Are the conclusions drawn adequately supported by the results?

Partly

Are sufficient details of methods and analysis provided to allow replication by others?

Partly

Reviewer Expertise:

Organized criminal groups in the African Sahel; Conflcit analysis; international humanitarian law

I confirm that I have read this submission and believe that I have an appropriate level of expertise to confirm that it is of an acceptable scientific standard, however I have significant reservations, as outlined above.

# Response

**Published:** 2026-08-27

**Authors:** Aitse John Zinna

**Affiliations:** Political Science and International Relations, Nile University of Nigeria, Abuja, Federal Capital Territory, Nigeria

We acknowledge your observations and have revised Section 3 accordingly. An integrating paragraph now opens the theoretical framework and establishes the logical architecture linking the three perspectives. While State Fragility Theory supplies the diagnostic component, explaining why the governance vacuum enabling banditry emerges. The Human Security and SSR paradigm supply the evaluative component, providing the standard against which government response is judged: not whether operations were conducted, but whether the security of ordinary people improved and was sustained.

 The Responsibility to Protect doctrine supplies the normative component, establishing the state's obligation to protect its population and the criteria against which that obligation is assessed. The three lenses therefore move from cause, to evaluation, to obligation. Each subsection now closes with an explicit, testable proposition (P1–P3) derived from the respective theory. Section 6.1 has been revised so that the theories generate and structure the analysis rather than describing it after the fact. We have also introduced Call's (2008) critique of the "failed state" concept in Section 3.1. This allows the study to specify a security gap in the state's constitutional obligation to protect life, rather than asserting wholesale state failure.   Section 3.2 now defines effectiveness explicitly as a three-dimensional construct: institutional coordination and consistency, community trust and perceived adequacy, and participatory and restorative delivery. Each dimension maps to the six survey items (Q13–Q18) used to measure it. This gives the study's principal dependent variable a stated conceptual basis before it is measured and distinguishes effectiveness as an outcome-oriented standard rather than a measure of activity.

 We thank you for these observations, which we believe have strengthened the study.

# Response

**Published:** 2026-08-31

**Authors:** Aitse John Zinna

**Affiliations:** Political Science and International Relations, Nile University of Nigeria, Abuja, Federal Capital Territory, Nigeria

We thank you for the generous assessment of the study's contribution, and in particular for recognising the value of the mixed-methods approach as a departure from the predominantly qualitative literature. We are grateful for the critical observations that follow, which we addressed. We have revised Section 3 accordingly. An integrating paragraph now opens the theoretical framework and establishes the logical architecture linking the three perspectives; State Fragility Theory supplies the diagnostic component, explaining why the governance vacuum enabling banditry emerges.

 The Human Security and SSR paradigm supplies the evaluative component, providing the standard against which government response is judged: not whether operations were conducted, but whether the security of ordinary people improved and was sustained. The Responsibility to Protect doctrine supplies the normative component, establishing the state's obligation to protect its population and the criteria against which that obligation is assessed. The three lenses therefore move from cause, to evaluation, to obligation. Each subsection now closes with an explicit, testable proposition (P1–P3) derived from the respective theory. Section 6.1 has been revised from confirmatory language to explicit proposition-testing language ("P1 is supported"), so that the theories generate and structure the analysis rather than describing it after the fact. We have also introduced Call's (2008) critique of the "failed state" concept in Section 3.1.

 This allows the study to specify a security gap in the state's constitutional obligation to protect life, rather than asserting wholesale state failure. Section 3.2 now defines effectiveness explicitly as a three-dimensional construct: institutional coordination and consistency, community trust and perceived adequacy, and participatory and restorative delivery. Each dimension maps to the six survey items (Q13–Q18) used to measure it. This gives the study's principal dependent variable a stated conceptual basis before it is measured, and distinguishes effectiveness as an outcome-oriented standard rather than a measure of activity. We are grateful for these observations, which have strengthened the study.
